# Meningitis

**Published:** 1877-08

**Authors:** C. A. Hentz

**Affiliations:** Quincy, Fla.


### MENINGITIS.

By C. A. HENTZ, M.D., Quincy, Fla.

Amongst the many communications on the subject of epidemic meningitis which have appeared in the journals, I have seen no suggestion made of a mode of treatment which I adopted some years ago, and which was attended with results so gratifying, that I have been anxious to see the suggestion thrown out to the medical profession, so that, if there is anything in it that may lead to a better management of this dreadful disease, suffering humanity may the sooner get the benefit of its adoption.

I have practiced through two malignant epidemic visitations of this disease; one in Jackson county, in this State, in 1857; and one in this place, Quincy, in January and February, 1870, and I became thoroughly disheartened with the utter uselessness of all efforts at cure in the usual routine way. Having observed that hot applications to the head were often grateful in cephalalgia from cold, I determined, during the prevalence of the last mentioned epidemic, to try them in meningitis.

The first case in which I made the experiment was that of a gentleman about fifty years of age, a merchant of this place, a gentleman of intelligence, and of rather a delicate constitution. He was suddenly and violently attacked with the most alarming symptoms; high fever, excruciating pain in the head, and still more severe about the middle of the spine; bloodshot eyes, scintillations of light, delirium coming on, etc., etc., symptoms so generally followed by insensibility and death. Having obtained the consent of the patient to the experiment, I proceeded at once to its energetic adoption; I first placed a sinapism to the spine, twelve inches wide, and reaching from the occiput to the sacrum, and let it stay; I had boiling water brought from the kitchen by the kettleful, and tempered with cold water to a degree of heat as great as he could possibly bear; had him supported by the shoulders over a tub placed by the bedside, and had this excessively hot water poured dipperful after dipperful, slowly and continuously over the head, for an hour or two, the patient meanwhile expressing a grateful sense of relief. I then had flannel petticoats wrung out of hot water, as hot as he could stand, wrapped round the head, and changed regularly, so as to sustain the heat continuously, for some twelve hours more. After this, as he improved, I substituted hot dry, for hot wet applications; winding up with simple dry flannel wrappings.

I gave internally, calomel in purgative doses till it operated well, and quinine in 20 grain doses, every two hours, till about two drachms were taken. It was well borne, and the case convalesced most favorably.

I did not have the opportunity to test this mode of practice in many cases, as the epidemic was on the wane, but in those where it was employed, the result was equally satisfactory. Not having notes of these cases, I cannot report them.

After unvarying failure with bleeding and cupping, leaching, blistering, ice, purging, etc., etc., the success attending this new departure in the way of treatment, has encouraged me to hope that in following out this suggestion, we can hereafter more successfully combat this terrible form of disease.

